# Genetic Differentiation in Hatchery and Stocked Populations of Sea Trout in the Southern Baltic: Selection Evidence at SNP Loci

**DOI:** 10.3390/genes11020184

**Published:** 2020-02-10

**Authors:** Rafał Bernaś, Anita Poćwierz-Kotus, Mariann Árnyasi, Matthew Peter Kent, Sigbjørn Lien, Roman Wenne

**Affiliations:** 1 Inland Fisheries Institute in Olsztyn, Department of Migratory Fishes, Rutki, 83-330 Żukowo, Poland; 2 Institute of Oceanology, Polish Academy of Sciences, 81-712 Sopot, Poland; anita@iopan.pl (A.P.-K.); RWenne@iopan.gda.pl (R.W.); 3 Centre for Integrative Genetics (CIGENE), Department of Animal and Aquacultural Sciences (IHA), Faculty of Life Sciences (BIOVIT), Norwegian University of Life Sciences (NMBU), 5003 Ås, Norway; mariann.arnyasi@nmbu.no (M.Á.); matthew.peter.kent@nmbu.no (M.P.K.); sigbjorn.lien@nmbu.no (S.L.)

**Keywords:** sea trout, SNP genotyping, stock composition

## Abstract

The impacts and interactions between hatchery-bred fish and wild fish populations has been a topic of active investigation in recent decades. In some instances, the benefits of stocking can be overshadowed by negative effects such as genetic introgression with natural populations, loss of genetic diversity, and dilution of local adaptations. Methods that facilitate the identification of stocked fish enable us to estimate not only the effectiveness of stocking but also the level of natural reproduction and the degree of hybridization. The longest Baltic river, the Vistula, also has the second highest discharge. Historically, it hosted numerous populations of the anadromous form of brown trout (sea trout); however, dam construction has since interfered with and reduced spawning migration to a rate that is much lower than before. Reduced spawning has resulted in a population collapse and a negative flow-on effect on commercial catches. In response, Poland (along with many other Baltic countries) initiated an intensive stocking program which continues today and which sees the average annual release of 700,000 smolts. As a consequence, today’s main-river and inshore catches come from stock-enhanced populations. High-throughput single-nucleotide polymorphism (SNP) genotyping was performed on samples of sea trout from southern Baltic populations; results suggest that a significant portion of the sea trout catches in the Vistula mouth region have direct hatchery origin and indicate the presence of Pomeranian specimens. SNP loci identified as outliers indicate a potential selection pressure that may be related with effects of hatchery breeding and mixing with natural populations. The brown trout SNP array applied in this study showed high effectiveness not only for population differentiation, but more importantly, it emerged as a sensitive tool to provide evidence of detection selection.

## 1. Introduction

Mixed-stock fisheries may target multiple anadromous fish populations; one strategy for documenting stock composition is genetic stock analysis [1]. This approach is routinely applied for determining migratory routes, timing, and catch share for several marine and anadromous fish [2], [3] and not only offers a way to perform stock-specific management but also facilitates the establishment of targeted stock-specific regulatory measures tailored towards safeguarding vulnerable populations [4]. In the Baltic Sea area, there is particular interest in learning more about the source(s) of the Atlantic salmon *Salmo salar* L. and brown trout *Salmo trutta* L. [5,6] caught by mixed stock fisheries. Generally, when fisheries operate close to a river mouth, the local river stock will be predominant in the catch; however the further the fisheries are from the river, the greater the extent of stock mixing [7].

Sea trout, the anadromous form of brown trout, resides as a native population in about 500 mainly small- and medium-sized rivers in the Baltic Sea region. In Poland, evidence suggest that 30 rivers have sea trout populations, and all of them have mixed status according to the Baltic Marine Environment Protection Commission (HELCOM) categorization [8]. The Vistula River is the longest river flowing into the Baltic Sea and has the second highest run-off after the Neva River [9]. In the past, large populations of sea trout have occupied the river with the main spawning ground located in the Carpathian area, about 1000 km from the Vistula river mouth in the Gdańsk Bay.

**Figure 1:**
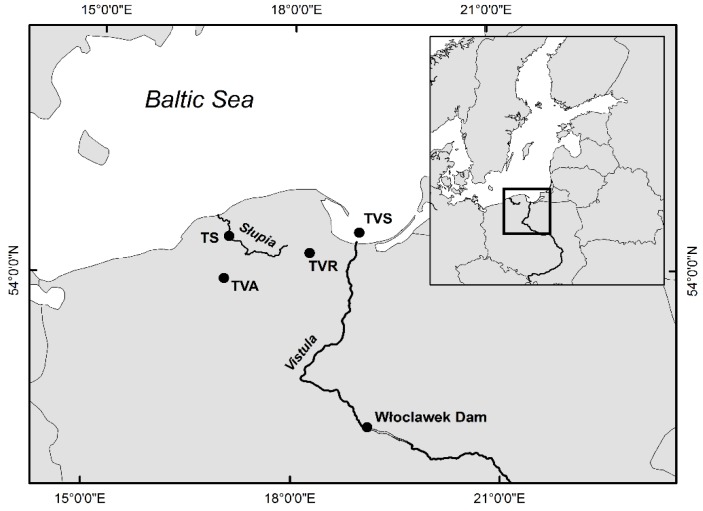
Map of the sites where sea trout were sampled in the Southern Baltic area. TS—Słupia River; TVS—Świbno area, TVR—Rutki hatchery, TVA—Aquamar hatchery.

Artificial stocking of the Vistula River began in the mid-nineteenth century. At that time, the majority of stockings used sea trout originating from spawners collected in the Dunajec River, about 800 km from the Vistula mouth and few other upper Vistula tributaries [10]; stocking was infrequent and irregular until the 1940s after which it became more stable. The main reason for population decrease seems to be dam constructions in the upper part of the basin, with the crucial date being 1968 when Włocławek Dam was built in the middle course and installed fishways proved to be insufficient (Figure 1). As one effect of this, the spawners were stopped below the dam, and from that time, the best reproductive area in the mountain tributaries was lost for many years. Since the construction of the dam in Włocławek was finished in 1969, the spawner collection in the upper segment of river was unsuccessful [11]. As a result, the new source of stocking material was established. Between 1972 and 1987, approximately 37% of sea trout used for enhancement was not Vistula sea trout and they originated from various Pomeranian rivers, mainly the Wieprza, Słupia, Łeba, Rega, and even the Parsęta or Łupawa rivers. This practice ended in 1985 when the location of spawner catches moved to the Vistula River estuary and suitable infrastructure was created [11,12]. Regular stocking has been performed since the 1960s and has seen a gradual increase in the number of individuals released. The volume of releases has been stably high since the 90s [13], and over the past 10 years, more than eight million smolts and 28 million younger stages (fry, alevins, and parr) have been released.

Since 1985, it has been assumed that the released material should represent progeny from individuals directly caught in the river during spawning migration. However, in practice, due to difficulties with spawner collection caused by seal predation, high water levels, etc., much of the roe used for production has originated from hatchery spawners belonging to a handful of broodstock from hatcheries in northern Poland. The biggest farm, Aquamar, maintains a hatchery stock established in the 1990s as a reserve stock for the Vistula River and is comprised of a putative winter sea trout stock. In subsequent years, this broodstock was supplemented by single winter sea trout specimens, and in the early 2000s, these fish began to deliver stocking material for release into the Vistula River. This hatchery population comprised 12–63% (average of 25%) of the material released annually into the Vistula River between 2004 and 2013 [11]. From the 1980s, a small proportion of the sea trout released originated from spawners held at the hatchery in Rutki (Department of Salmonid Fish Breeding, Inland Fisheries Institute). This broodstock was a mix of sea trout collected in the Vistula mouth and sea trout from the River Dalälven in Sweden imported in 1998. The Dalälven brood fishes were the progeny of the Vistula sea trout that was introduced there in the 1960s. While both the Aquamar and Rutki hatchery lines have been used for Vistula stocking over the last 15 years (Figure 1), the majority of material is from Aquamar, with only 2–5% of total stocking coming from Rutki. At the present time, also progeny from hatchery stock in Dąbie are used for Vistula stocking. The Dąbie broodstock was established in 2006 based on sea trout collected in the Vistula mouth. From 2013, these fish comprised 15 to 45% (average of 30%) of the sea trout released. In 2015, the old fishway was rebuilt, and in 2015–2016, about 2000 sea trout migrated upstream [14]. Actual spawning grounds exist mainly in the Drwęca River and, in a few small tributaries below Włocławek, also in several short stretches of bigger rivers flowing into the lower Vistula that are available for migration.

Tagging studies exploring sea trout costal migrations have shown that individuals from smolt stocking which were released close to the mouths of the Pomeranian rivers had very high levels of straying individuals, which can explain a significant share of Pomeranian genotypes among sea trout collected in the Vistula firth area [15,16]. Results from mass tagging experiments conducted in the 1970s in the Vistula River showed that Vistula sea trout were concentrated in the southern Baltic, mainly near the Gdańsk Bay and the Vistula mouth, which is where 65% of sea trout whitling tag returns were obtained [16].

Relative to wild stocks, salmonid hatchery fish often display a reduced performance and survival rate [17] and display weaker homing instincts [15]. Experiments have also shown that stocking a target river with hatchery fish whose parents come from that particular rivers wild population can be more successful that stocking with fish whose genetics have developed in rivers located elsewhere [18]. This reduced fitness is the result of genetic, morphological, physiological, ecological, and behavioral changes induced in the hatchery environment [19]. Unintentional selection in the captive-rearing process and reduced natural selection seem to be the mechanisms driving this maladaptation to the wild environment [20].

Several studies sought to develop tools to genetically differentiate between southern Baltic Sea populations, mainly from the Pomeranian and Vistula rivers and local hatchery stocks. However, none using allozymes [21], mitochondrial DNA (mtDNA) [22], and microsatellites (short tandem repeats (STR)) [23,24] have reported diagnostic markers. In contrast, the application of a small number of SNP markers (identified as polymorphic in Polish sea trout populations genotyped on a SNP microarray) have provided promising results [25]. Genotyping a time series of sea trout samples from six Polish rivers with these SNP markers has revealed temporal changes in genetic composition for *S. trutta* populations in the Vistula River that were related to differential stocking strategies [26]. Lack of temporal stability in genetic composition has also been reported in a recent study utilizing microsatellites [27]. Until now, no genetic study (based on allozymes, mtDNA, STR, or low number of SNPs) has fully resolved the issue of the share of hatchery genotypes and the presence of the Pomeranian lines in sea trout from the Vistula firth area. An SNP array robustly testing thousands of loci may be better able to report subtle differences in genomic variation [28] and may reveal evidence of mutations subject to evolutionary selective forces affecting protein function or gene expression levels [29].

The main goal of the present study was to differentiate hatchery origin and Pomeranian genotypes in sea trout collected in the south of the Gulf of Gdańsk, a region representing the Vistula population. In addition, the genotyping of a significant number of SNP loci provided an opportunity to identify loci under selection and to highlight potential effects of breeding operations (e.g., faster growth and warm water). Additionally, temporal genetic stability in a population from the Słupia River was tested in order to demonstrate the effect of genetic drift caused by artificial spawning using a small number of parental combinations.

## 2. Materials and Methods

### 2.1. Sampling and Genomic DNA Isolation

Sea trout samples from the Słupia River were collected in autumn 2008 (TS8; N = 18) and 2009 (TS9; N = 25) from adult fish caught in trap about 30 km from the river mouth in Słupsk City, 54.460538°, 17.039682°, during spawning migration. Sampled fish represented both sexes and were at sea age from 1 to 3+ years. Adult specimens collected in the Vistula mouth area (TVS; N = 28; southern International Council for the Exploration of the Sea (ICES) 26 area) were harvested using gillnets in October 2009 by fishermen during several trials over a few fishing grounds located on fishing square T4. Sea trout were mainly female and at sea age from 1 to 3+ years. The other two samples originated from hatcheries. The first (TVR; N = 21) was collected from smolts in 2009 taken from a brood stock created and maintained in the Department of Salmonid Fish Breeding at the Inland Fisheries Institute in Rutki. The second investigated hatchery line (TVA; N = 19) was sampled in 2009 from adults in the hatchery farm Aquamar in Miastko. Fin clips were stored in 95% EtOH solution at 4 °C. Genomic DNA was extracted using Qiagen DNeasy 96 blood and tissue kit, as recommended by the manufacturer. DNA samples were analyzed in 1.0% agarose gels and quantified on a NanoDrop device.

### 2.2. SNP Genotyping and SNP Validation

Samples were genotyped using a custom-developed brown trout (*Salmo trutta*) llumina iSelect SNP microarray at the Centre for Integrative Genetics (CIGENE), Norway, as in Linløkken et al. [30]. Although the array contained assays for 5509 SNP loci, a significant proportion (n = 1570) was excluded from analysis because of one or more of the following reasons: (i) cluster patterns suggested multisite or paralogous site variants; (ii) evidence for a nearby variant (null allele) affecting genotype assignment; and (iii) the SNP was monomorphic or mitochondrial. An acceptable threshold of missing data across samples was determined to be 85%, leading to the removal of four SNPs; an additional 101 with minor allele frequencies (MAF) less than 0.01 were also discarded. After filtering, data from a set of 3843 SNPs that were polymorphic in our samples were analyzed. The mean percent of missing data per individual was 0.078 and varied between 0 and 3.79 (SD 0.444) (Table S1).

### 2.3. Data Analysis

The number of polymorphic loci, the mean number of alleles, and the observed and expected heterozygosity (*H*_O_ and *H*_E_) for all loci and for each population were estimated in Arlequin 3.5.2.2 software (University of Berne, Bern, Switzerland) [31]. Departures from the Hardy–Weinberg equilibrium were also tested using the Markov Chain Monte Carlo random algorithm in Arlequin 3.5.2.2; population-specific *F*_IS_, pairwise weighted *F*_ST,_ and overall F-statistic were estimated by analysis of molecular variance (AMOVA). A sequential Bonferroni type method was employed to correct for multiple significance tests. Clustering of sea trout populations was calculated using the Structure 2.3.4 software (Stanford University, Stanford, CA, USA) [32] assuming K = 1 to 6. The STRUCTURE algorithm included the admixture model and correlated allele frequencies. Five iterations of each K were performed with 200,000 Markov Chain Monte Carlo (MCMC) repetitions and 100,000 burn-ins. Two methods were used to infer the number of clusters present in the dataset: first was the K value with highest ln Pr(X|K) [32]; second was the Evanno method (∆K) [33] in Structure Harvester v0.6.94 [34]. The CLUMPP 1.1.2 software (Stanford University, Stanford, CA, USA) was used to find the best alignment of the results from the five replicate cluster analyses [35].

Genetic structure was also examined by Principal Coordinates Analysis (PCoA) done in GenAlex 6.502 (Australian National University, Acton, Australia) [36]. The POPTREE2 program (Kagawa University, Kagawa, Japan) was used to build a neighbor joining tree using D_A_ distances [37], with 1000 bootstrap replications [38]. To determine the most likely origin of all 111 sea trout specimens, individual self-assignment tests using partial Bayesian method [39] implemented in GeneClass2 (INRA/CIRAD, Montpellier, France) were conducted (assignment threshold of score = 0.05) [40]. The hierarchical island model with 100,000 simulations implemented in Arlequin was used to spot outlier SNPs. SNPs that had *F*_ST_ values outside the 99% quantile that were based on coalescent simulations were considered as candidates for diversifying selection [41]. Outlier detection performed in Arlequin was validated by more restrictive method in Bayescan 2.1 (University of Berne, Bern, Switzerland) [42]. Settings with samples size 5000, thinning interval 10, pilot runs 20, pilot run length 5000, and 100,000 additional burn-in were used. Outliers were identified using a false discovery rate (*q*-value) threshold of 0.05. For new SNP panels based on calculated outlier loci, the overall *F*_ST_ and pairwise comparisons were recalculated similar to Bayesian clustering in STRUCTURE. The identification of selection signatures can help to understand the genetic factors and patterns engaged in important biological traits because these regions might have adaptive and functional relevance underlying their selection [43,44]. Putative outliers were assigned to their chromosomal position conferring to the Atlantic salmon linkage map, which contains approximately 5000 SNPs and was constructed using genotyping data from 143 families comprised of 3297 fish. This map contained 29 linkage groups, each of which was assigned to its specific chromosome [45,46].

Blast2go (BioBam Bioinformatics, Valencia, Spain) [47] was used to obtain the Gene Ontology (GO) annotation. Homology searching was first realized through a BLAST search of the available flanking sequences for each SNP on the The National Center for Biotechnology Information (NCBI) public database (accessed on: 6 April 2018). Blast2go then retrieved GO terms associated with the BLAST hits. For sequences without information via NCBI, an Ensembl (accessed on: 11.05.2018) genome annotation pipeline was applied with searches against all available fish species [48]. These short-range disequilibria have practical interest. They are the basis for association mapping of genes that contribute to, e.g., phenotypes. Blocks of unrecombined chromosome can also be exploited to identify recent and ongoing selective sweeps [49,50]. A linkage disequilibrium (LD) was tested between pairs of loci using the Arlequin 3.5.2.2 software. The exact linkage disequilibrium *p*-values for the observed allelic association under the null hypothesis of random allelic assortment were done by Markov Chain Monte Carlo with 10,000 steps and 1000 dememorizations. Additionally, LD as *r*^2^ or D’ values was calculated in Multiallelic Interallelic Disequilibrium Analysis Software (MIDAS) [51] and TASSEL 5.2.37 (Biotechnology Bldg, Ithaca, NY, USA) [52]. To build the linkage map, genetic position for all flanking sequences of the outlier loci was determined on Atlantic salmon genome assembly (ICSASGv2; NCBI accession GCF 000233375 [46] and was analyzed in TASSEL.

## 3. Results

### 3.1. Genetic Polymorphism and Diversity

The mean number of alleles in investigated stocks of individuals varied between 1.906 and 1.961 (Table 1). The lowest values were found for hatchery lines TVR and TVA, 1.906 and 1.927, respectively. Observed heterozygosity was also lowest in those stocks, similar to the number of polymorphic loci (Table 1). The highest number of loci with significant departures from the Hardy–Weinberg equilibrium was found in specimens that originated from hatcheries.

Population-specific F_IS_ were insignificant (*p* < 0.05) in all stocks. Analysis of molecular variance was performed for three scenarios: “All samples” (TS9 + TS8 + TVS + TVR + TVA); “Sampled in wild” (TS8 + TS9 + TVS); and “Hatcheries” (TVR + TVA). In all scenarios, the largest amount of variance had its source in within-population diversity (Table 2).

However, large differences in overall *F*_ST_ were observed between analysed scenarios. For “All samples”, overall genetic differentiation (*F*_ST_) was equal to 0.033, which indicates a low level of differentiation. In the tests performed for sea trout sampled in the wild, overall *F*_ST_ was even lower and reached 0.019. The highest general *F*_ST_ was observed for hatchery fish and reached 0.062. The dissimilarity of the hatchery lines, especially from the TVR samples, is well presented by pairwise comparisons. All tests were significant (*p* < 0.05) (Table 3). The highest values of *F*_ST_ were indicated for the TVR vs. TVA comparison. A small genetic distance was observed between both samples collected from the Słupia River, which might indicate low temporal stability in that population. 

### 3.2. Genetic Structure and Relationships

**Figure 2:**
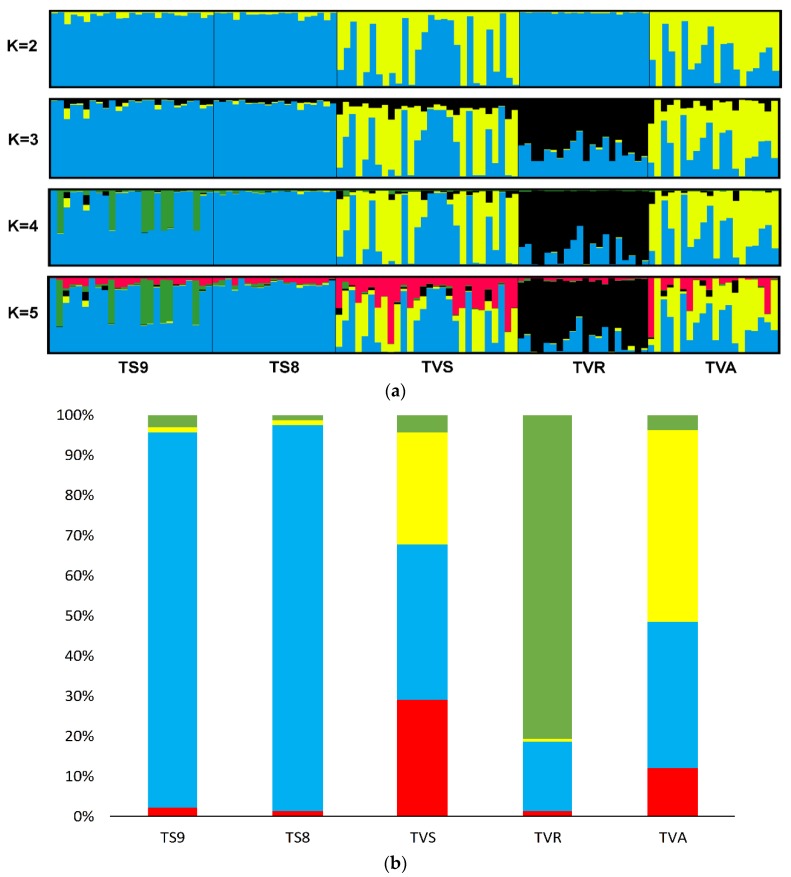
(**a**) Population structure of Vistula sea trout based on Bayesian clustering analysis for the set of all loci: Computations from 5 independent runs were treated in CLUMPP 1.1.2 and plotted with DISTRUCT 1.1. Each line corresponds to an individual. (**b**) Proportion of membership of each predefined population in each of the 4 clusters estimated in STRUCTURE.

Bayesian estimation of genetic structure and individual membership performed in STRUCTURE 2.3.4 indicated that the maximum value of Δκ was for K = 4 (ΔK = 140.86). Ln Pr(X|K) slowly increased from K = 1 to K = 4, and ΔK presented a sharp peak for K = 4 (Figure S1). In this scenario, samples from the Słupia River TS8 and TS9 comprised a common cluster. Sea trout from the Vistula mouth TVS and from hatchery lines TVR and TVA had own clusters. The composition of the sample from the Vistula mouth area included the share of Pomeranian genotypes and a large number of genotypes represented by the Aquamar hatchery clad (TVA) (Figure 2a,b).

Other scenarios have lower ΔK values. The scenario with K=2 (ΔK = 37.80) grouped TS population with TVS and Aquamar hatchery TVA. For K = 3 ΔK = 3.04, and first clad has TS samples, the second clad comprised TVS and TVA, but TVR has own distinct cluster. The last scenario with K = 5 shows separate clusters for every investigated stock.

**Figure 3:**
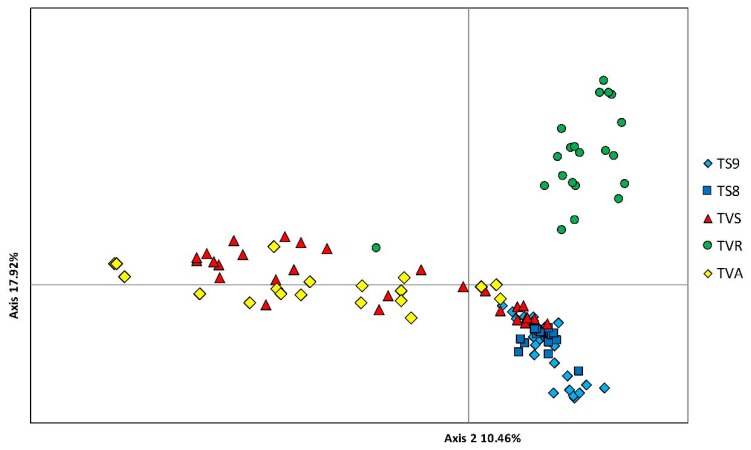
Principal coordinates analysis (PCoA) based on all single-nucleotide polymorphisms (SNPs).

Results from Principal Coordinates Analysis (PCoA) are in agreement with Bayesian clustering. The PCoA plot shows large differences between TVR and other samples. Additionally, the significant share of Pomeranian genotypes in the sample from the Vistula mouth was confirmed (Figure 3).

For better presentation of patterns of the genetic compositions inside studied stocks, the individual self-assignment test was performed (Table 4). Results show that the mentioned share of Pomeranian genotypes between sea trout harvested in the Vistula mouth area reached 28.58% (both TS8 and TS9). The component originating from the Aquamar hatchery had 10.71% inside TVS samples and TVS component amounted to 20% inside TVA. The TVR individuals showed high homogeneity without representation in other stocks.

Genetic relationships between investigated sea trout samples obtained from the phylogenetic analysis showed three clads with high bootstrap support. A neighbor-joining (NJ) tree (Figure S2) grouped individuals from the Słupia River into one clad (TV8 and TV9). The next clad comprised specimens sampled in the Vistula mouth area (TVS) and hatchery line from Aquamar (TVA). The third clad included individuals from the Rutki hatchery line (TVR).

### 3.3. Analysis of Putative Outliers and Linkage Disequilibrium

**Figure 4:**
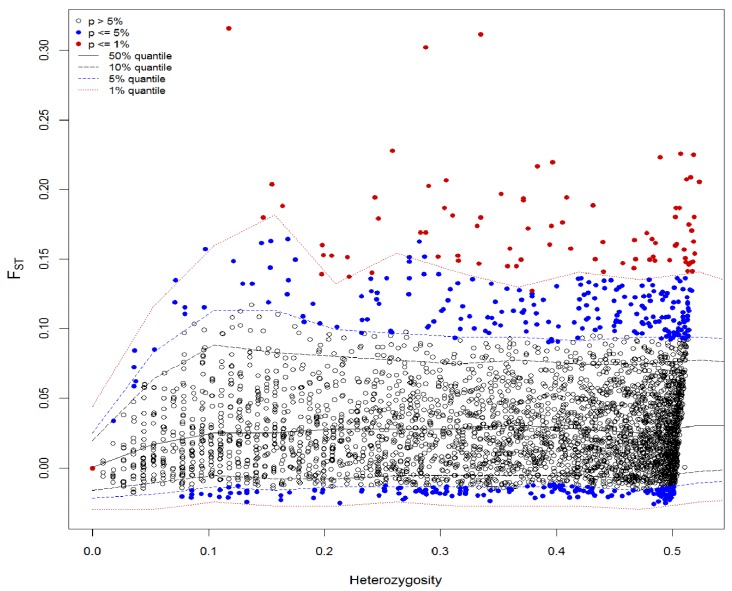
Analysis of outlier SNPs using a hierarchical model: SNPs that are above the 99% quantile of the simulation model were considered as SNPs under potential selection. SNPs above the upper solid line were considered as candidates for divergent selection, and those below the lower solid line were considered as candidates for balancing selection. SNPs that are between the dashed blue lines are neutral.

**Figure 5:**
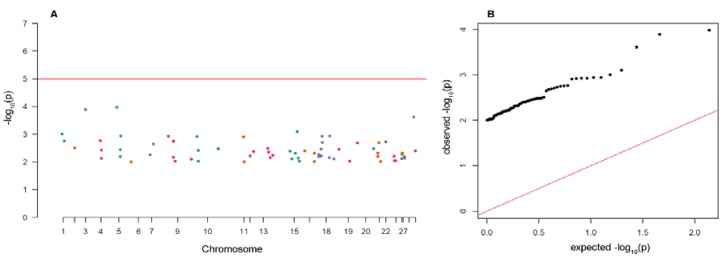
Manhattan (**A**) and Q-Q plot (**B**) builds for 86 putative outlier loci detected with the Arlequin and Bayescan methods: Each dot on this figure corresponds to a SNP within the dataset, while the horizontal red line denotes the genome-wide significance. The Manhattan plot contains −log10 observed *p*-values for putative outlier SNPs (y-axis) plotted against their corresponding position on each chromosome (x-axis), while the Q-Q plot contains expected −log10-transformed *p*-values plotted against observed −log10-transformed *p*-values.

Eighty-three SNPs were classified as potential outliers in Arlequin 3.5.2.2 (Figure 4) and 29 were classified in Bayescan 2.1 (Figure S3). The majority (n = 25) of outliers detected by the Bayesian approach was also detected by Arlequin and can be considered as strong outliers (Table S3). All detected loci were on a positive (divergent) selection. The distribution of the outlier loci across chromosomes are displayed on a Manhattan plot constructed for all 86 putative outliers (Figure 5). Global *F*_ST_ from AMOVA performed only for 83 candidate loci detected in Arlequin reached 0.172 and was evidently higher than the global *F*_ST_ calculated for the 3843 polymorphic loci (0.033) for *p* = 0.001. Pairwise *F*_ST_ also showed much higher values, even between samples from consequent years in the Słupia River (Table S4). Results for Bayesian estimation of genetic structure based on 83 outlier loci performed in STRUCTURE 2.3.4 indicated that the maximum value of ΔK was for K = 2 (ΔK = 700.07). The scenario with K = 2 grouped TS population with fish from the Vistula mouth TVS and hatchery line TVA. Rutki hatchery TVR has own cluster (Figure S4).

Atlantic salmon chromosome 18 (Ssa18) includes the greatest number of markers showing evidence of selection (n = 12); followed by Ssa15 (n = 9) and Ssa09 (n = 7); and then Ssa05, Ssa10, and Ssa13 (n = 5 for all) (Table S2). No outlier was located on chromosomes Ssa8, Ssa17, and Ssa25. Gene ontology specified from BLAST (X, N, or BLAT) database searches (Table S3) showed wide spectrum of genes coding. For 12 outliers without annotations for genus *Salmo,* annotation information was obtained using other fish species, especially from Atlantic cod (*Gadus mohua*), which has been the subject of extensive sequencing and annotation. During scoring, many orthologs among annotated genes were generally observed between close evolutionary species from genus *Oncorhynchus* and from the *Esocidae* family, e.g., LOC106573934 in the *Salmo salar*, LOC109889095 for *Oncorhynchus kisutch,* and 105011366 (sgta) for *Esox lucius,* all coding glutamine-rich tetratricopeptide repeat-containing protein alpha-like.

**Figure 6:**
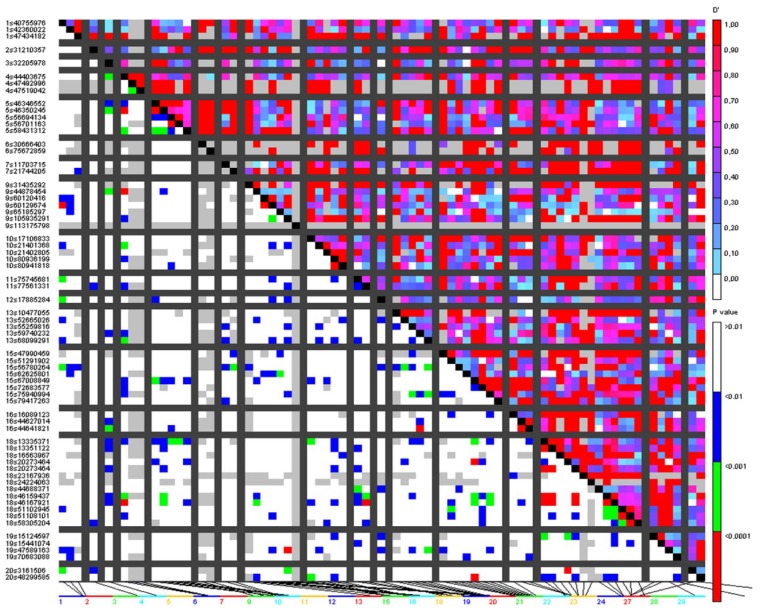
Heatmap representing variation in linkage disequilibrium (LD) on outlier loci distributed on *Salmo salar* chromosomes: The markers were presented on the x and y axes according to their chromosomal positions. The D’ values are denoted by a color scale from white (0.0) to dark red (1.0) in the upper triangle. The *p*-values ranging from nonsignificant (>0.01; white) to highly significant (<0.0001; red) are shown in the lower triangle.

To detect potentially linked regions with signs of selection, the linkage disequilibrium tests were performed for outlier loci. Simulations performed in Arlequin between pairs of loci in particular stock did not confirm the null hypothesis of no linkage disequilibrium for outlier loci combinations tested. The highest number of significantly linked pairs was detected for loci SalHit:S8517710121 (44 links), Gdist:S2685113092 (42 links), and cDNA:S363339747 (41 links) (Table S2). Four analyzed stocks of individuals show similar levels of detected links (between 310 and 364), except for TVS sample where 590 links were scored (Table S2). For 83 candidate loci distributed according BLAST matching on 29 *Salmo salar* chromosomes, we also tested 3404 (full matrix) possible linkage combinations in TASSEL. Results show 305 links with *p* < 0.01 with D’ values ranging between 0.44 and 1 (mean 0.80) and *r*^2^ values ranging between 0.12 and 1 (mean 0.34). Similar to the Arlequin method, the highest number of significantly linked loci were located on chromosome 18 and on loci Gdist:S541107 1153 and Gdist:S99221 1923 (Table S2 and Figure 6).

## 4. Discussion

### 4.1. Stocking History and Evidence From Alternative Non-Genetic Methods

In this study, a 5.5 K Illumina SNP iSelect genotyping array developed for brown trout (*S. trutta*) was used to analyze the genetic relationship between sea trout specimens from the southern Baltic Sea. The experiment explored the proportion of Pomeranian and hatchery origin genotypes among sea trout harvested in the southern Gulf of Gdańsk in the vicinity of the Vistula River mouth. In rivers flowing to the investigated area, regular stocking with sea trout smolts, fry, and alevins has been conducted from the 1960s [8]. From 2000–2013, approximately 35% of the individuals used for Vistula River stocking were from hatcheries, with stocking using material gathered from river spawners reaching about 65% (Inland Fisheries Institute in Olsztyn, unpublished data). Results from assignment tests showed that the share of the Aquamar hatchery genotypes among samples from costal catches around the Vistula mouth (TVS) reached approximately 11%. Moreover, in TVS samples, the presence of Pomeranian genotypes was at a level of 28%. This level of admixture stock should be considered high compared with studies based on scale readings where, from similar areas to TVS, the presence of the Pomeranian scale traits was not detected in samples [54]. Considering the fact that TVS sample collection was performed during October, the presence of the Pomeranian genotype can indicate on its significant share in the Vistula River stock. As Degerman et al. [55] showed in their study, it is possible that straying of mature fish from August to November may be related to spawning. However, that phenomenon varies considerably between the rivers. The same high variation between rivers has been described for Pomeranian rivers as well [15]. At this point, the genetic analysis is congruent with tagging experiments.

### 4.2. Vistulian Clade and the Breeding Lines

Genetic structure (Pritchard test, PCA) and NJ tree show that, among adult specimens harvested near the Vistula mouth (TVS sample), the Aquamar TVA hatchery line represents a significant share; roughly 30%. The comparison of share of the Pomeranian genotypes between TVS and TVA samples suggest that the real proportion of Pomeranian genotypes is lower because it is represented by a significant proportion in the TVA hatchery stock (37%). However, results from PCA analysis do not confirm this. Further, they show that some specimens from Aquamar are only close to Pomeranian genotypes where TVS partially overlap them. Bayesian analysis of genetic structure also shows the presence of another clade; it can be called the “Vistulian” clade. The highest share of these genotypes occurred within the TVS sample. This is important information indicating that some level of natural spawning in the Vistula Basin occurs. Despite the percent of Vistula clade in the Aquamar sample being lower than that in the TVS sample, it still exists in the hatchery line. The dissimilarity of samples from the TVR hatchery shows that sea trout imported from Dalälven are relatively distant (genetically) from contemporary Polish populations. The reason for this could be genetic drift during years of hatchery breeding. The theory that this line is close to the original Vistulian population from before the Włocławek Dam is unlikely, especially if we consider high genetic distance between present Polish populations and Dalälven. Furthermore, recent results from microsatellite analysis of the historical and contemporary Vistulian populations show lower genetic distance between them [27].

Analysis of the samples from the Słupia River was performed not only as a comparison with the Vistula line but also for temporal stability test. There was an assumption that artificial spawning performed annually on this river has a strong influence on genetic structures in sea trout populations [56]. The estimated level of genetic differentiation between sea trout from the Słupia River years 2008 and 2009 shows small but significant differences in what can occur in populations located in unstable environments. The population number estimated by fish counter in this river is largely variable, with great differences between seasons. In 2008, the number of spawners reached more than 7000, whereas in 2009, it only reached 3500 [14].

### 4.3. Outlier Loci and Potential Selection Pressure

Outlier analysis showed differences in the total number of detected loci between methods. The difference in the number of candidate markers could be either due to the inability of the Bayescan methods to detect signatures of selection at these loci or because a large number of the outlier loci detected by Arlequin 3.5 are false positives (type I error) [57]. However, the Arlequin method is also modified to reduce the number of false positive outlier loci compared to original algorithm (n-island model vs. hierarchical island model) [41]. Noteworthy is that loci detected in both methods overlap by as much as 86 percent.

Detected putative outlier loci indicated the existence of selection pressure among investigated sea trout stocks that can be related to processes existing in breeding lines. Generally, about 2% (1% for Bayesian method) of the analyzed loci were classified as outliers, a portion that has been reflected in other studies (e.g., Gutierrez et al. [44] found 1% within wild and hatchery lines of *Salmo salar* investigated using a custom 6.5K SNP array). In the study designed for discrimination of different classes of hybrid between wild Atlantic salmon and aquaculture escapees, where a 220K SNP array was used, the level of outliers was also reported at about 1% (analysis with Bayesian approach) [32]. However, in a more recent study (using the same array have used in this paper), a pairwise Arlequin analysis indicated that 4.5–7.0% of bi-allelic loci were outliers [30]. A similar percentage (3%) was calculated in a study of sea trout populations originated mainly from the Schleswig-Holstein area (Baltic and North Sea coast) [58]. The available annotation information showed that, for those outlier SNPs located within genes, most of these genes were related to general cellular, metabolic, and biological regulation. However, a number was also related to systems and processes that may be significant from a selection perspective. For example, several are involved in cell–matrix and cell–cell adhesion interactions (Gdist:S96636_9765, Gdist:S96636_14657, and Gdist:S96636_14046) and collagen alpha chains (Gdist:S56626 7438). Some others are associated with ubiquitin-activating enzymes (cDNA:S480496_654, ESTNV 30276_856, Gdist:S153878_3763, and Gdist:S153878_9142); insulin-like growth factor 1 (Gdist:S49874_6547); tyrosine phosphatase; receptor type T (Gdist:S625346_1946); and Ras suppressor protein 1 gene which encodes a protein that is involved in the Ras signal transduction pathway, growth inhibition, and nerve-growth factor-induced differentiation processes [59] (loci Gdist:S314969_4922, Gdist:S186377_2059, and SalHit:S126835_16266). The potential effects for some of them in salmonids were already described, and the regulation of these functions have also proven to be reflected in the origin of fish and their life history, e.g., genetically based differences in expression of the insulin-like growth factor 1 are related to anthropogenic selection pressures in salmon and trout [60] and collagen alpha 1 showed consistently higher expression in stressed hatchery salmon [61]. However, it should be noted that gene annotations were performed using the most recent *Salmo salar* assembly [46]; despite brown trout being the closest relative of the Atlantic salmon, there are significant differences between the species. For instance, the difference in chromosome number between *S. salar* (2n = 54–58 [62]) and *S. trutta* (2n = 80 [63]) indicates extensive chromosomal rearrangements [64], mainly in the form of chromosome fusions in Atlantic salmon [46,65]. The net nucleotide divergence between *S. salar* and *S. trutta* was estimated in the study by Leitwein et al. [64] as 1.87%, which indicates a relatively recent divergence time between these two closely related species. Analysis of linkage disequilibrium performed for outlier loci shows that higher linkage occurs between the loci located on chromosome 18, which is congruent with the distribution of detected outlier loci. Detected putative outlier loci and regions under selection shows differences between analyzed stocks. That samples from the mixed fishery from Gdańsk Bay had almost double the number of linked outlier loci than other single stock indicates that fish from hatcheries and from wild populations are subjected to different selection pressures. This could be related with life histories, local adaptations, or domestication effects. This evidence should be subject for further investigation with emphasis to hatchery specific traits (faster growth, warm water adaptation, or health issues) and their influences for next generations performance.

## 5. Conclusions

Obtained results have revealed several facts that may prove important from a management perspective. The share of the hatchery origin specimens inhabiting the Vistula mouth area is high, which can be indicative of relatively good stocking effectiveness. The breeding line from Aquamar is genetically similar to the Pomeranian fish. The discovered “Vistulian” clad indicates some level of natural reproduction among the Vistula population. The analysis also showed disorder in genetic temporal stability within the population from the Słupia River, which is related to stocking practices and significant population fluctuations. The brown trout SNP array applied in this study showed high effectiveness not only for population differentiation but also, more importantly, as a sensitive tool for evidence of detection selection. Obtained outliers exposed that, in investigated sea trout populations, significant evidence for signatures of selection occurs and many candidate markers are linked with molecular functions related to the selection process of specific traits.

## Figures and Tables

**Table 1:** Genetic diversity for five sea trout stocks from the southern Baltic Sea. N—number of individuals, NPL—number of polymorphic loci, MNA—mean number of alleles, *H*_O_—observed heterozygosity, *H*_E_—expected heterozygosity, DHWE—loci with deviation from H–W equilibrium after Bonferroni correction a population specific *F*_IS._

Stock	N	NPL	MNA	Ho	He	DHWE	F_IS_
**TS9**	25	3706	1.949	0.339	0.324	4	−0.034
**TS8**	18	3657	1.931	0.332	0.319	7	−0.011
**TVS**	28	3780	1.961	0.345	0.333	3	−0.024
**TVR**	21	3560	1.906	0.328	0.314	12	−0.019
**TVA**	19	3640	1.927	0.326	0.324	13	0.020

**Table 2:** Analysis of Molecular Variance (AMOVA) applying the F_ST_ estimator of Weir and Cockerham [53] variance component, calculated for 3 scenarios: all samples, specimens sampled from wild, and only hatchery stocks.

	Among Populations		Within Populations	
	Variance component	% variation	Variance component	% variation
All samples	22.74	3.40	646.83	96.60
Sampled in wild	13.31	1.98	655.90	98.01
Hatchery stocks	42.49	6.19	643.54	93.80

**Table 3:** Below diagonal: F_ST_ values for pairwise comparisons of 5 sea trout stocks. All values were significant for a *p* = 0.05; on diagonal: average number of pairwise differences within population; and above diagonal: Nei’s genetic distance *D*_A._

Stock	TS9	TS8	TVS	TVR	TVA
**TS9**	1281.257	6.032	33.938	64.040	51.076
**TS8**	0.004	1292.351	34.579	64.132	50.736
**TVS**	0.025	0.025	1316.761	67.770	15.800
**TVR**	0.047	0.047	0.049	1255.973	84.902
**TVA**	0.037	0.037	0.011	0.061	1315.641

**Table 4:** Results of the individual self-assignment test computed using GeneClass2 software [40]: Results are presented with percent score of most likely source (threshold *p* < 0.05).

Stock	TS9	TS8	TVS	TVR	TVA
TS9	56.02	43.98	0.00	0.00	0.00
TS8	52.63	47.37	0.00	0.00	0.00
TVS	17.41	11.17	60.71	0.00	10.71
TVR	0.00	0.00	0.00	100.00	0.00
TVA	0.00	0.00	20.00	0.00	80.00

## Supplementary Materials

The following are available online at https://www.mdpi.com/2073-4425/11/2/184/s1, Table S1: Missing data per individual, Figure S1: Best ΔK; Table S2: Results from pairwise linkage disequilibrium test performed in Arlequin, Figure S2: A neighbor-joining tree, Table S3: Detected outliers loci; Figure S3: F_ST_ outlier analysis of 3843 SNPs in BAYESCAN 2.1., Table S4: F_ST_ values for pairwise comparisons of 5 sea trout stocks based on 83 outlier loci, Figure S4: Population structure of Vistula sea trout based on Bayesian clustering analysis for the set of 83 outlier loci.
