# Supplementary material for: Genetic Differentiation in Hatchery and Stocked Populations of Sea Trout in the Southern Baltic: Selection Evidence at SNP Loci
Source: Genes (Basel). 2020 Feb 10;11(2):184. doi: 10.3390/genes11020184 (PMC7073890; doi:10.3390/genes11020184)
Supplement: Supplementary file 1 [file genes-11-00184-s001.zip › Supplementary data/Figure S3.pdf]

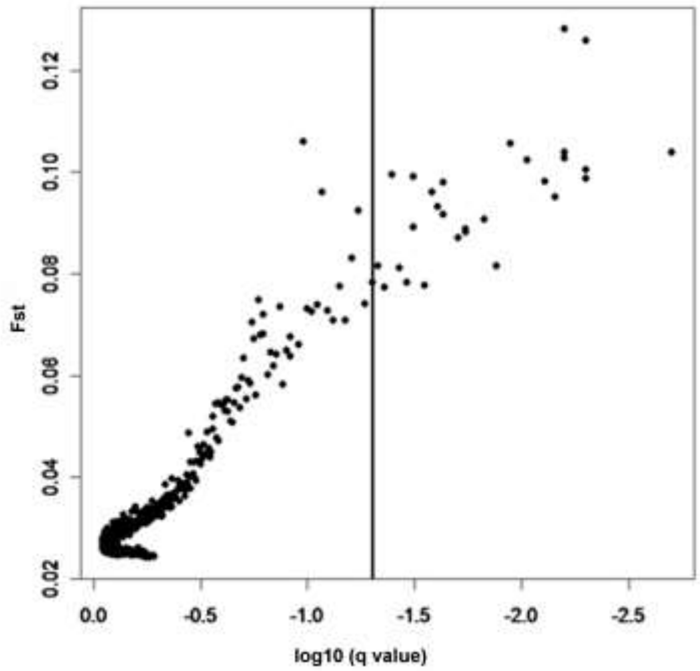

Figure S3. F_{ST} outlier analysis of 3843 SNPs in BAYESCAN 2.1. Pairwise F_{ST} values are plotted against the log10 transformed q-values (the minimum false discovery rate at which a locus becomes significant).
