# Supplementary material for: Genetic Differentiation in Hatchery and Stocked Populations of Sea Trout in the Southern Baltic: Selection Evidence at SNP Loci
Source: Genes (Basel). 2020 Feb 10;11(2):184. doi: 10.3390/genes11020184 (PMC7073890; doi:10.3390/genes11020184)
Supplement: Supplementary file 1 [file genes-11-00184-s001.zip › Supplementary data/Table S2.docx]

Table S2. Results from pairwise linkage disequilibrium test performed in Arlequin.

| **SNP name** | **Chromosome** | **TS9** | **TS8** | **TVS** | **TVR** | **TVA** | **∑** |
| --- | --- | --- | --- | --- | --- | --- | --- |
| Gdist:S377637_7617 | *ssa01* | 3 | 4 | 12 | 3 | 4 | 26 |
| SalHit:S98813_6560 | *ssa01* | 5 | 5 | 2 | 3 | 0 | 15 |
| cDNA:S353186_3074 | *ssa02* | 4 | 5 | 2 | 7 | 4 | 22 |
| Gdist:S18807_5080 | *ssa03* | 3 | 10 | 16 | 3 | 2 | 34 |
| cDNA:S165871_3361 | *ssa04* | 3 | 1 | 9 | 12 | 7 | 32 |
| cDNA:S267203_6159 | *ssa04* | 2 | 4 | 3 | 5 | 3 | 17 |
| cDNA:S47753_3153 | *ssa04* | 4 | 4 | 4 | 4 | 3 | 19 |
| Gdist:S268511_3092 | *ssa05* | 6 | 7 | 18 | 5 | 6 | 42 |
| Gdist:S268511_6812 | *ssa05* | 8 | 6 | 3 | 6 | 6 | 29 |
| SalHit:S500022_1016 | *ssa05* | 11 | 2 | 1 | 1 | 6 | 21 |
| SalHit:S88367_2537 | *ssa05* | 4 | 4 | 5 | 7 | 6 | 26 |
| SalHit:S88367_9559 | *ssa05* | 2 | 3 | 7 | 6 | 4 | 22 |
| SalarSNP:GCR_cBin14108_Ctg1_271 | *ssa06* | 0 | 0 | 0 | 6 | 0 | 6 |
| cDNA:S621834_5067 | *ssa07* | 5 | 5 | 1 | 5 | 2 | 18 |
| SalHit:S618840_2758 | *ssa07* | 3 | 2 | 16 | 7 | 0 | 28 |
| cDNA:S195837_3240 | *ssa09* | 2 | 6 | 2 | 6 | 3 | 19 |
| cDNA:S302429_3381 | *ssa09* | 0 | 0 | 6 | 5 | 0 | 11 |
| cDNA:S363339_747 | *ssa09* | 4 | 1 | 19 | 10 | 7 | 41 |
| Gdist:S2588_1936 | *ssa09* | 5 | 3 | 17 | 4 | 3 | 32 |
| Gdist:S2588_9839 | *ssa09* | 3 | 1 | 14 | 8 | 4 | 30 |
| Gdist:S422914_5157 | *ssa09* | 2 | 1 | 0 | 7 | 0 | 10 |
| SalHit:S85177_10121 | *ssa09* | 4 | 6 | 19 | 8 | 7 | 44 |
| Gdist:S186377_2059 | *ssa10* | 1 | 4 | 4 | 0 | 1 | 10 |
| Gdist:S49874_6547 | *ssa10* | 1 | 4 | 3 | 2 | 8 | 18 |
| Gdist:S49874_900 | *ssa10* | 4 | 5 | 12 | 5 | 9 | 35 |
| SalarSNP:ESTNV_30276_856 | *ssa10* | 0 | 3 | 4 | 0 | 8 | 15 |
| SalHit:S102823_1006 | *ssa10* | 11 | 3 | 2 | 4 | 1 | 21 |
| cDNA:S21271_1377 | *ssa11* | 1 | 8 | 5 | 7 | 7 | 28 |
| cDNA:S495556_576 | *ssa11* | 12 | 4 | 8 | 0 | 8 | 32 |
| Gdist:S112887_7924 | *ssa11* | 5 | 4 | 6 | 5 | 1 | 21 |
| Gdist:S352552_6424 | *ssa12* | 3 | 3 | 16 | 4 | 5 | 31 |
| cDNA:S480496_654 | *ssa13* | 1 | 2 | 1 | 7 | 4 | 15 |
| cDNA:S675938_3404 | *ssa13* | 11 | 3 | 6 | 1 | 2 | 23 |
| Gdist:S115607_10942 | *ssa13* | 10 | 3 | 4 | 3 | 1 | 21 |
| Gdist:S220636_231 | *ssa13* | 10 | 6 | 3 | 5 | 5 | 29 |
| LD:S110750_883 | *ssa13* | 0 | 6 | 4 | 4 | 4 | 18 |
| Gdist:S8065_3229 | *ssa14* | 4 | 4 | 12 | 5 | 0 | 25 |
| cDNA:S363729_1681 | *ssa15* | 8 | 0 | 2 | 3 | 1 | 14 |
| cDNA:S404555_12194 | *ssa15* | 3 | 1 | 2 | 3 | 3 | 12 |
| cDNA:S77853_1811 | *ssa15* | 6 | 1 | 18 | 2 | 4 | 31 |
| Gdist:S150832_4425 | *ssa15* | 2 | 4 | 9 | 2 | 1 | 18 |
| Gdist:S17128_3728 | *ssa15* | 2 | 2 | 3 | 5 | 4 | 16 |
| Gdist:S455907_4238 | *ssa15* | 12 | 2 | 13 | 0 | 3 | 30 |
| Gdist:S52502_2377 | *ssa15* | 11 | 3 | 6 | 1 | 4 | 25 |
| Gdist:S69367_3982 | *ssa15* | 4 | 8 | 16 | 8 | 2 | 38 |
| SalarSNP:GCR_cBin18664_Ctg1_166 | *ssa15* | 5 | 7 | 13 | 2 | 1 | 28 |
| SalarSNP:GCR_cBin34489_Ctg1_38 | *ssa16* | 3 | 4 | 3 | 7 | 4 | 21 |
| SalHit:S116298_7643 | *ssa16* | 3 | 2 | 5 | 4 | 6 | 20 |
| SalHit:S256514_3653 | *ssa16* | 3 | 4 | 6 | 5 | 3 | 21 |
| cDNA:S611416_1700 | *ssa18* | 3 | 1 | 6 | 0 | 2 | 12 |
| Gdist:S115813_12050 | *ssa18* | 3 | 3 | 4 | 5 | 0 | 15 |
| Gdist:S153878_3763 | *ssa18* | 4 | 9 | 6 | 3 | 2 | 24 |
| Gdist:S153878_9142 | *ssa18* | 3 | 9 | 6 | 2 | 2 | 22 |
| Gdist:S245672_3136 | *ssa18* | 3 | 3 | 8 | 2 | 3 | 19 |
| Gdist:S541107_1153 | *ssa18* | 3 | 2 | 11 | 5 | 6 | 27 |
| Gdist:S9002_10672 | *ssa18* | 2 | 3 | 3 | 6 | 11 | 25 |
| Gdist:S96636_14046 | *ssa18* | 9 | 7 | 4 | 4 | 8 | 32 |
| Gdist:S96636_14657 | *ssa18* | 6 | 6 | 6 | 4 | 6 | 28 |
| Gdist:S96636_9765 | *ssa18* | 7 | 6 | 5 | 5 | 7 | 30 |
| Gdist:S99221_1923 | *ssa18* | 2 | 4 | 8 | 8 | 2 | 24 |
| SalHit:S324937_1374 | *ssa18* | 10 | 0 | 6 | 0 | 3 | 19 |
| Gdist:S185339_7770 | *ssa19* | 2 | 3 | 7 | 6 | 4 | 22 |
| Gdist:S208396_4887 | *ssa19* | 2 | 9 | 8 | 5 | 2 | 26 |
| Gdist:S314969_4922 | *ssa19* | 3 | 4 | 7 | 2 | 6 | 22 |
| Gdist:S88922_2932 | *ssa19* | 2 | 0 | 6 | 1 | 0 | 9 |
| cDNA:C124697205_473 | *ssa20* | 2 | 6 | 4 | 4 | 4 | 20 |
| SalHit:S19196_949 | *ssa20* | 1 | 2 | 6 | 1 | 2 | 12 |
| Gdist:S104994_4018 | *ssa21* | 6 | 10 | 3 | 7 | 6 | 32 |
| Gdist:S386025_2013 | *ssa21* | 13 | 2 | 16 | 1 | 5 | 37 |
| Gdist:S386025_9401 | *ssa21* | 6 | 1 | 15 | 0 | 4 | 26 |
| Gdist:S56626_7438 | *ssa21* | 4 | 1 | 8 | 5 | 9 | 27 |
| cDNA:S467031_884 | *ssa22* | 2 | 5 | 11 | 7 | 0 | 25 |
| Gdist:S101169_6923 | *ssa23* | 5 | 12 | 5 | 3 | 5 | 30 |
| Gdist:S250809_7698 | *ssa23* | 11 | 1 | 4 | 8 | 0 | 24 |
| Gdist:S830668_2442 | *ssa23* | 4 | 3 | 2 | 1 | 1 | 11 |
| LD:S38447_13299 | *ssa23* | 3 | 2 | 3 | 2 | 2 | 12 |
| Gdist:S137894_1019 | *ssa24* | 2 | 3 | 17 | 3 | 7 | 32 |
| Gdist:S35568_4383 | *ssa24* | 6 | 3 | 6 | 7 | 3 | 25 |
| Gdist:S232219_5301 | *ssa27* | 3 | 5 | 5 | 3 | 5 | 21 |
| SalHit:S126835_16266 | *ssa27* | 3 | 3 | 7 | 9 | 3 | 25 |
| cDNA:S297048_3936 | *ssa28* | 2 | 4 | 2 | 9 | 4 | 21 |
| Gdist:S13053_6942 | *ssa28* | 6 | 6 | 1 | 4 | 7 | 24 |
| Gdist:S625346_1946 | *ssa29* | 2 | 2 | 12 | 0 | 2 | 18 |
|  |  | 364 | 320 | 590 | 354 | 310 | ∑ |
