# Supplementary material for: Genetic Differentiation in Hatchery and Stocked Populations of Sea Trout in the Southern Baltic: Selection Evidence at SNP Loci
Source: Genes (Basel). 2020 Feb 10;11(2):184. doi: 10.3390/genes11020184 (PMC7073890; doi:10.3390/genes11020184)
Supplement: Supplementary file 1 [file genes-11-00184-s001.zip › Supplementary data/Table S4.docx]

**Table S4.** *F*_ST_ values for pairwise comparisons of 5 sea trout stocks based on 83 outlier loci. All values were significant for a p=0.05.

| **Stock** | **TS9** | **TS8** | **TVS** | **TVR** | **TVA** |
| --- | --- | --- | --- | --- | --- |
| **TS9** | 24.390 | 0.417 | 3.533 | 6.596 | 6.222 |
| **TS8** | 0.017 | 24.710 | 3.811 | 6.960 | 6.574 |
| **TVS** | 0.115 | 0.123 | 27.470 | 9.688 | 1.295 |
| **TVR** | 0.203 | 0.213 | 0.258 | 26.930 | 12.363 |
| **TVA** | 0.190 | 0.200 | 0.046 | 0.308 | 27.680 |
