# Supplementary figures and images for: Genetic Differentiation in Hatchery and Stocked Populations of Sea Trout in the Southern Baltic: Selection Evidence at SNP Loci
Source: Genes (Basel). 2020 Feb 10;11(2):184. doi: 10.3390/genes11020184 (PMC7073890; doi:10.3390/genes11020184)

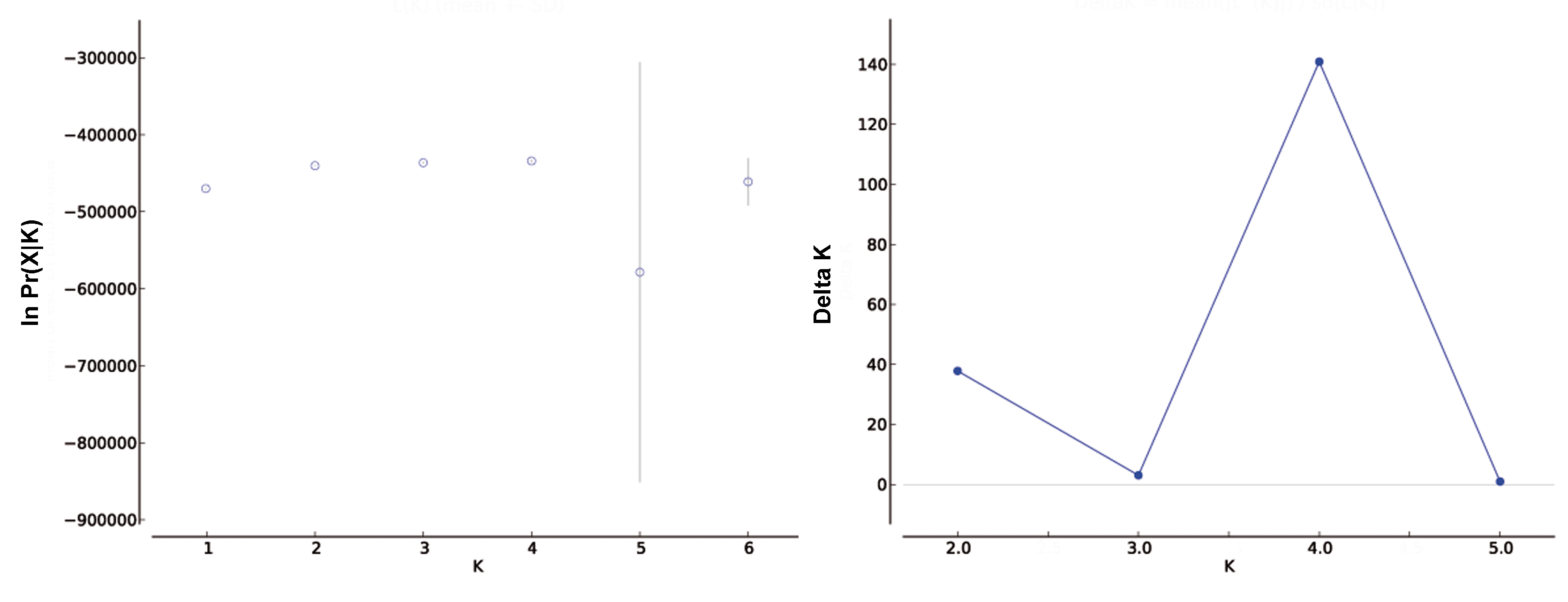

Supplement: Supplementary file 1 [file genes-11-00184-s001.zip › Supplementary data/Figure S1.tif]
